# The structure of KRAS^G12C^ bound to divarasib highlights features of potent switch-II pocket engagement

**DOI:** 10.1080/21541248.2025.2505441

**Published:** 2025-05-20

**Authors:** Micah C. Fernando, Gregory B. Craven, Kevan M. Shokat

**Affiliations:** a Department of Cellular and Molecular Pharmacology and Howard Hughes Medical Institute, University of California, San Francisco, CA, USA; b Department of Cellular and Molecular Pharmacology, University of California, San Francisco, CA, USA; c Department of Chemistry, University of California, Berkeley, CA, USA

**Keywords:** KRAS, oncogene, divarasib, structure, inhibitor

## Abstract

KRAS is the most frequently mutated oncogene in human cancer. In multiple types of cancer, a missense mutation at codon 12 substitutes a glycine for a cysteine, causing hyperactivation of the GTPase and enhanced MAPK signalling. Recent drug discovery efforts culminating from work during the past decade have resulted in two FDA-approved inhibitors, sotorasib and adagrasib, which target the KRAS^G12C^ mutant allele. Ongoing medicinal chemistry efforts across academia and industry have continued developing more potent and efficacious KRAS^G12C^ inhibitors. One agent in late-stage clinical trials, divarasib, has demonstrated robust overall response rates, in some cases greater than currently approved agents. Divarasib also exhibits enhanced covalent target engagement *in vitro* and significant specificity for KRAS^G12C^, yet the structural details of its binding have not been published. Here we report a high-resolution crystal structure of cysteine-light KRAS-4B^G12C^ in complex with divarasib. Though it binds in the same allosteric pocket as sotorasib and adagrasib, the switch-II loop in each crystal structure takes on a distinct conformation differing as much as 5.6 Å between the Cα atom of residue 65 with sotorasib. Additionally, we highlight structural features of the drug complex that may guide future medicinal chemistry efforts targeting various *KRAS* alleles.

## Introduction

Small GTPases such as KRAS are composed of a G-domain and a hypervariable region that cycle between an inactive (GDP-bound) and active (GTP-bound) conformation [1]. The G-domain is responsible for binding nucleotide and coordinating effector binding. Hotspot mutations in the P-loop of the G-domain such as *KRAS*^*G12V*^, *KRAS*^*G12D*^, and *KRAS*^*G12C*^ perturb the normal equilibrium by inhibiting GTP hydrolysis and locking KRAS in its active conformation [1,2]. Though *KRAS*^*G12C*^ is the third most common allele, initial studies focused on taking advantage of the nucleophilic cysteine handle to trap KRAS^G12C^ in its inactive state [3].

Two FDA-approved therapies, sotorasib and adagrasib, which covalently modify the GDP-bound state of KRAS^G12C^ were approved in 2021 and 2022, respectively [4,5]. These two first-in-class agents demonstrated the druggability of KRAS almost four decades after it was first identified as a human oncogene [6]. Three additional covalent KRAS^G12C^ inhibitors currently undergoing clinical trials (olomorasib-LY3537982, opnurasib-JDQ443, and divarasib-GDC-6036) have also reported encouraging clinical outcomes [7,8]. More recently, the development of BI-0474 also targeting the inactive KRAS^G12C^ state was disclosed, demonstrating potent *in vitro* engagement and good pharmacodynamics [9]. Co-crystal structures of advanced KRAS^G12C^ drugs have facilitated the development of next-generation inhibitors as well as molecules targeting other alleles, yet not all the structures are available in the public domain. Specifically, divarasib is currently in clinical trials for NSCLC and has resulted in the highest overall response rate of up to 56.4%, yet its co-crystal structure coordinates have not been published or deposited in the PDB [10,11].

Improved understanding of KRAS^G12C^ engagement via this cryptic pocket has accelerated medicinal chemistry efforts and resulted in numerous lead compounds. We became particularly interested in the bound structure of divarasib because of its emergence in a chemical genetic screen in our laboratory demonstrating the presence of the switch-II pocket across the small GTPase family [12]. Here we present a 1.90 Å resolution crystal structure of KRAS^G12C, C51S, C80S, C118S^ bound to divarasib. Examination of the structure revealed key residues important for binding and activation of the covalent inhibitor in the pocket. Notably, we observed some key differences between this structure and other published structures of GDP-bound KRAS^G12C^ complexed to adagrasib, sotorasib, opnurasib, or BI-0474. These comparisons highlight the diversity in covalent switch-II pocket engagement and could advance our understanding of distinct drug binding modes.

## Results

**Figure 1.**
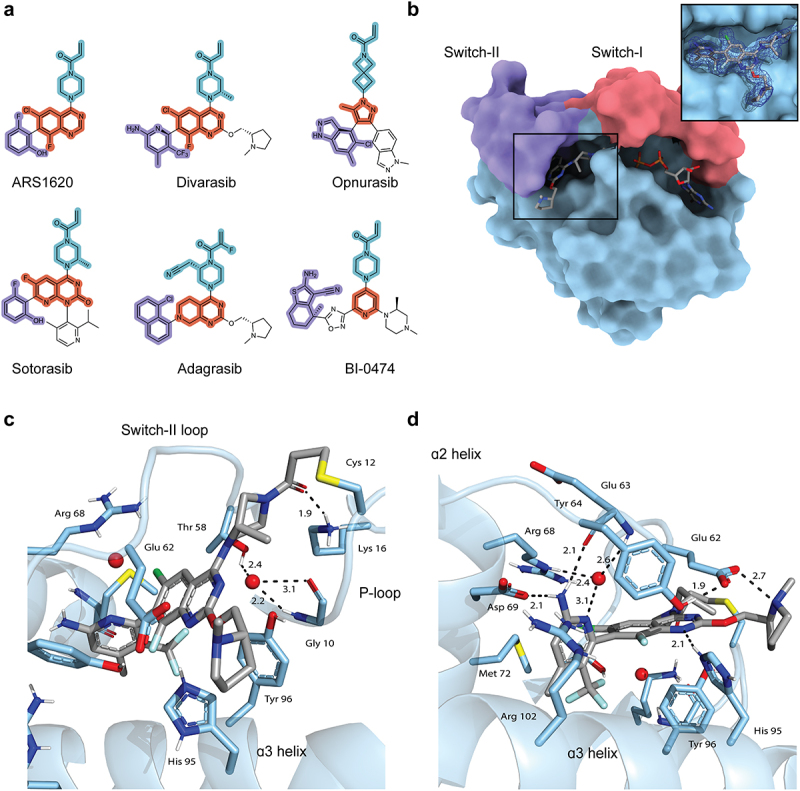
(a) Structures of compounds targeting inactive KRAS^G12C^. The amide-linked warhead is shown in blue, the core is in red, and the buried R group is in purple. (b) Surface representation of the KRAS^G12C^ ● divarasib complex (PDB code: 9DMM) with the switch regions colored. Crystallographic information is provided in Table SI. The zoomed-in view of the ligand has residues 61–64 of the switch-II loop hidden to visualize the back of the pocket. The 2Fo-fc map of divarasib is contoured to 1σ. (c, d) Two different views of divarasib in the binding pocket. Distances are in Å. Hydrogens are added for clarity.

The KRAS^G12C^ drug candidate, ARS-1620, demonstrated *in vivo* tumour growth inhibition for the first time, verifying the potential of KRAS-targeted therapy [13]. It established the effectiveness of a quinazoline core while maintaining the acrylamide warhead from compound 12 [14]. Extensive medicinal chemistry efforts from Amgen, Mirati Therapeutics (BMS), and Genentech (Roche) derivatized the core to optimize *in vitro* potency and overall bioavailability. Each of these molecules engages the inactive state of KRAS^G12C^ via the allosteric switch-II pocket in a slightly different orientation, taking advantage of the dynamic environment. Novartis relied on *in silico* optimization and structure-based design to develop opnurasib, employing a distinct mono-cyclic scaffold and amide linker while still utilizing the acrylamide warhead (Figure 1a) [15]. Boehringer Ingelheim in collaboration with the Fesik group took advantage of an initial reversible fragment screen and structure-based design to ultimately design BI-0474 (Figure 1a) [9].

The structure of the GDP-bound KRAS^G12C^ ● divarasib complex was determined at 1.90 Å by replicating the crystallization conditions for sotorasib (Figure 1b). Following molecular replacement with the apoprotein, electron density in the switch-II pocket indicated an adduct with cysteine 12 with an interatomic distance of 1.83 Å being consistent with a carbon-cysteine bond. Apart from partial density for the Gln 61 sidechain, the switch-II loop is well defined (Figure S2). Though Glu 63’s carboxyl group has elevated B-factors near 50 Å^2^, residues of the switch-II loop have only moderate B-factors with the average between residues 58 and 76 being 33.5 Å^2^.

Divarasib forms several key interactions in the switch-II pocket of KRAS^G12C^. In the back of the pocket, Lys 16 is positioned 1.9 Å from the carbonyl of the acrylamide to form a hydrogen bond (Figure 1c). Tyr 96 sits behind the core to form the hydrophobic base of the pocket (Figure 1c). Interestingly, a water molecule is present in a network with Thr 58 and Gly 10 and not displaced by the methylpiperazine (Figure 1c). This water is also conserved in a structure of an adagrasib precursor, prompting the addition of a cyanomethyl group to the final compound 3.3 Å from the Gly 10 backbone (Figure S1B) [4].

Divarasib extends into the pocket forming interactions with the switch-II loop and α2 helix. The quinazoline core of divarasib is positioned to form a hydrogen bond with His 95 while Tyr 64 and Glu 62 extend from the switch-II loop to form a lid (Figure 1d). In the back of the pocket, a second water molecule forms a network with Glu 63, Arg 68 and the pyridine’s tertiary amine (Figure 1d). The pyridine also coordinates with Asp 69 and Glu 63 via its primary amine at distances of 2.1 Å (Figure 1d).

**Figure 2.**
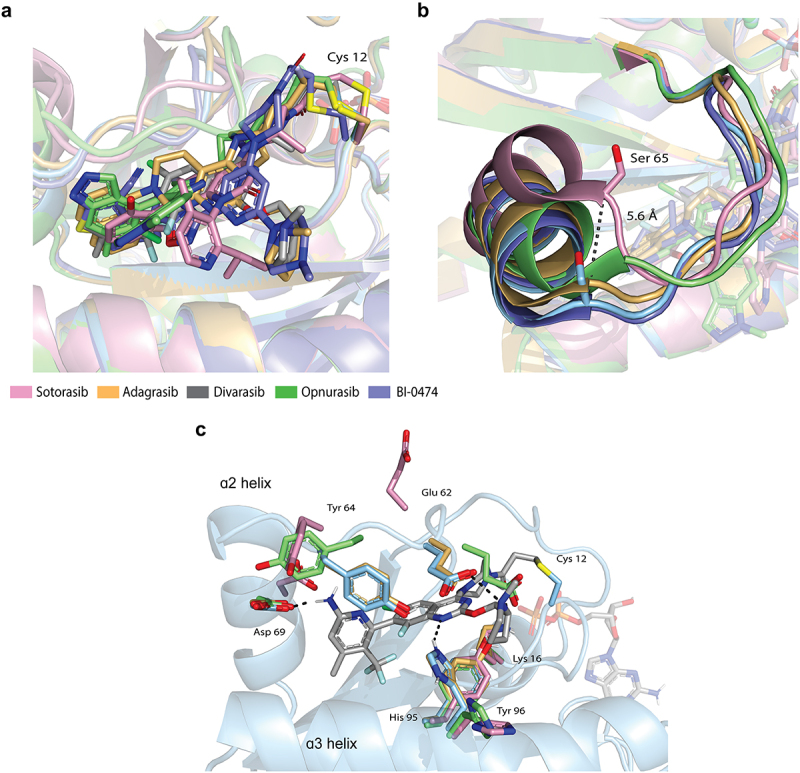
(a) Overlay of each KRAS^G12C^ co-crystal structure depicting sotorasib in pink (PDB: 6OIM), adagrasib in orange (PDB: 6UT0), opnurasib in green (PDB: 7R0M), BI-0474 in purple (PDB: 8AFB), and divarasib in blue. (b) Depiction of the switch-II loop and α2 overlaid in each complex. Black dashed line represents the distance between Cα at Ser 65 of the sotorasib and divarasib structure. (c) Binding site of divarasib depicting sidechains Lys 16, Glu 62, Tyr 64, Asp 69, His 95, Tyr 96, from each structure.

The mono- or bi- cyclic cores of each inhibitor align well in the crystal structures though differences exist between how the R groups engage with the pocket (Figures 2a and S1). In the solvent-exposed region, divarasib and adagrasib take advantage of the same methylpyrrolidine substituent, however, sotorasib differs with only a carbonyl at this position (Figure 1d). The isopropyl-methylpyridine off the N1 position of sotorasib is reported to bind in a novel His 95-groove defined by its flipped-out conformation (Figure S1A) [5]. Compared to ARS-1620, this allows more depth to the pocket and increases van der Waals interactions. Opnurasib’s indazole binds in a similar region with His 95 oriented away from the drug towards the solvent (Figure S1C). In contrast, divarasib and adagrasib both take advantage of the protonated N3 of His 95 for hydrogen bonding to the core (Figures 2c and S1B). Accordingly, acquired resistance mechanisms against treatment with adagrasib include missense mutations such as H95D/Q/R^7^. In the crystal structure of BI-0474, His 95 also points inward, though it is further from the pyridine core and instead interacts with Tyr 64 (Figure S1D) [9].

The switch-II loop itself varies between the structures as well. The loops are well defined in each of the crystal structures except for partial density often seen for the Glu 61 sidechain. The most striking observation is the distance between the Cαs at Ser 65 which is 5.6 Å in the sotorasib and divarasib structures (Figure 2b). Similar to His 95, Glu 62 is flipped out and away from sotorasib, whereas it makes an interaction with the methylpyrrolidine of divarasib and adagrasib (Figures 2c and S1B). Additionally, Tyr 64 is flipped out in the sotorasib and opnurasib structures but encloses the pocket with the other drugs.

Notably, the interaction of the acrylamide carbonyl with Lys 16 is conserved among the four drugs (Figures S1 and 2c). This residue has been proposed to activate the ligand for 1,4 addition by the thiolate [16]. The lysine is positioned in each structure to stabilize the transition state oxyanion and forms a hydrogen bond with the carbonyl in the crystal structures (Figures 2c and S1).

The lipophilic pocket at the back of the switch-II pocket supports hydrophobic packing (Figure 1b,d). Divarasib’s pyridine substituent contains a CF_3_ group, while sotorasib employs a fluorine, and adagrasib utilizes an 8-chloronaphthyl that positions chlorine to fill this sub-pocket (Figure 1a). Notably, the synthesis of divarasib isolated the active atropisomer to position the pyridine perpendicular to the core for the CF_3_ group to fill this sub-pocket, much like the use of the *S*-atropisomer of ARS-1620 [13,17]. On the other hand, BI-0474’s unique benzothiophene substituent is linked to the core via an oxadiazole to reach into this sub-pocket (Figure S1D).

Opnurasib was optimized from structure-based *de novo* screen and exhibits a slightly different binding mode [15]. It is pushed up against the wall of the switch-II loop to contact residues along the loop and α2 helix such as Ser 65 and Asp 69 while avoiding interaction with Tyr 96 and His 95 (Figure S1). In contrast, divarasib reaches to make an interaction with Asp 69 while maintaining a hydrogen bond to His 95; a unique combination not seen in the other drug complexes (Figures 1d and S1).

## Discussion

Potent state-selective switch-II pocket inhibitors have revolutionized the treatment of KRAS^G12C^-driven tumours [3]. Structural and functional characterization of KRAS^G12C^ inhibitors have accelerated our ability to generate next-generation therapeutics. In 2022, the development of divarasib was announced by Genentech (Roche) at the AACR meeting with significantly improved *in vitro* and *in vivo* potency compared to ARS-1620 [11]. In this work, we report a crystal structure of GDP-bound KRAS^G12C^ in complex with divarasib and highlight elements of switch-II pocket engagement. The drug makes interactions throughout the switch-II pocket, taking advantage of both polar and hydrophobic contacts such as His 95 and Asp 69.

Furthermore, the switch-II pocket varies among each complex and suggests varied engagement of the region (Movie S1). However, inferences from crystal structures are limited to comparisons between static structures captured in a lattice and do not fully represent the physiological state of the protein. Crystal contacts between monomers can also affect the protein’s conformation, though divarasib’s switch-II, α2, and α3 helix region do not directly participate in these contacts. Additionally, different buffer conditions were used to obtain opnurasib, adagrasib, and BI-0474 co-crystal structures which may affect the final conformation of the protein complex.

## Materials and methods

### Protein expression and purification

The *KRAS*^*G12C*^ DNA sequence encoding the human Cys-light allele (residues 1–169) was codon optimized, synthesized, and cloned into the pProEx vector by GenScript. KRAS^G12C^ protein was purified using previously published methods [12]. Briefly, a colony from transformed BL21(DE3) cells grown on LB agar plates was used to inoculate terrific broth containing 50 µg/mL carbenicillin. When the optical density reached 0.6 at 37ºC, the culture temperature was reduced to 18ºC, and protein expression was induced by the addition of IPTG to 1 mm. After 16 h at 18ºC, the cells were pelleted by centrifugation (19,000 × g, 25 min) and lysed in lysis buffer [20 mm Tris 8.0, 500 mm NaCl, 5 mm imidazole] with EDTA-free protease inhibitor (Sigma-Aldrich) using ultrasonication (40% duty cycle for 3 × 3 min cycles). The lysate was clarified by high-speed centrifugation (19,000 × g, 15 min). The supernatant was incubated with Co-TALON resin (Clonetech, Takara Bio USA, 2 mL slurry/litre culture) at 4ºC for 1 h with constant end-to-end mixing. After washing with lysis buffer (30 mL/litre culture), the protein was eluted with elution buffer [20 mm Tris 8.0, 300 mm NaCl, 250 mm imidazole]. To this protein solution was added 0.05 mg of His-tagged TEV protease (Berkeley QB3 MacroLab) and 1 mg of GDP per mg of protein. The mixture was dialysed against TEV Cleavage Buffer [20 mm Tris 8.0, 300 mm NaCl, 5 mm imidazole, 1 mm EDTA, 1 mm DTT] at 4 ºC using a 10K MWCO dialysis cassette (Fisher Scientific) until LC-MS analysis showed full cleavage (typically 16–24 hr.). MgCl_2_ was added to a final concentration of 5 mm and the mixture was incubated with 2 mL Ni-NTA (Qiagen) beads at 4 ºC for 1 h to remove residual His-tagged proteins and peptides. The protein was concentrated using a 10K MWCO centrifugal concentrator (Amicon-15, Millipore) to a volume of 1 mL and purified by size exclusion chromatography on a Superdex 75 10/300 GL column using SEC buffer [20 mm HEPES 7.5, 150 mm NaCl, 1 mm MgCl_2_] (GE Healthcare Life Sciences). Fractions were pooled for crystallography and stored at − 78ºC.

### Crystallography

Divarasib (MedChemExpress) was added to 6 mg of pure protein as a 50 mm solution in DMSO. The mixture was allowed to stand at 23ºC until LC-MS analysis of the reaction mixture showed full conversion to a single covalent adduct (19951 Da). The reaction mixture was purified by size exclusion chromatography (Superdex75, 20 mm HEPES 7.5, 150 mm NaCl, 1 mm MgCl_2_) and concentrated to 60 mg/mL using a 10K MWCO centrifugal concentrator (Amicon-15, Millipore). Crystals were grown at 20ºC in a 96-well plate utilizing hanging-drop vapour diffusion. Crystals grew in the previously established condition for the KRAS^G12C^ ∙ sotorasib complex (1 mm MgCl_2_, 0.1 M MES pH 6.5, 30% PEG4000) and then were transferred to a cryoprotectant solution composed of the well solution supplemented with 20% ethylene glycol and flash-frozen in liquid nitrogen. The dataset was collected with a Pilatus3 6 M 100 hz detector and a wavelength of 1.02978 Å at 100 K at the Advanced Light Source beamline 2.0.1. The dataset was indexed and integrated using iMosflm and determined by molecular replacement using Phaser in the PHENIX software suite [18–20]. The crystal structure of GDP-bound K-Ras(G12C)-sotorasib adduct (PDB 6OIM) was used as the initial model. The structure was manually refined with Coot and PHENIX [21]. Data collection and refinement statistics are listed in Table S1. Structural visualization was carried out in PyMOL and ChimeraX [22,23].

## Supplementary Material

KRAS_Divarasib_report_SI_changes clean file.docx

## Data Availability

The atomic coordinates and structure factors for KRAS^G12C^ bound to divarasib reported in this study are openly available in the Protein Data Bank (PDB) at accession code: 9DMM. Other structures referenced in this report include PDB: 6OIM, 6UT0, 8AFB, and 7R0M. Movie SI showing the morphing from the bound structures of sotorasib to divarasib is available at https://figshare.com/ doi: 10.6084/m9.figshare.28586384.
