# Supplementary material for: The structure of KRASG12C bound to divarasib highlights features of potent switch-II pocket engagement
Source: Small GTPases. 2025 May 20;15(1):1–7. doi: 10.1080/21541248.2025.2505441 (PMC12101598; doi:10.1080/21541248.2025.2505441)
Supplement: KRAS_Divarasib_report_SI_changes clean file.docx [file KSGT_A_2505441_SM2839.docx]

**Data Collection**

Resolution range 25.62 - 1.9 (2.05 - 1.9)

Space group P 21 21 21

Cell dimensions

*a, b ,c* (Å) 45.89, 59.63, 61.75

*α, β, γ* (º) 90, 90, 90

Total reflections 84269 (17065)

Unique reflections 13733 (2691)

Multiplicity 6.1 (6.3)

Completeness (%) 98.25 (97.57)

Mean I/sigma (I) 5.25 (3.05)

Wilson B-factor 18.87

R-merge 0.2952 (0.9228)

R-meas 0.3246 (1.018)

R-pim 0.1316 (0.4155)

CC1/2 0.931 (0.627)

CC* 0.982 (0.878)

**Refinement Statistics**

Reflections used in refinement 13642 (2654)

Reflections used for R-free 659 (116)

R-work 0.1700 (0.1709)

R-free 0.2279 (0.2384)

Number of non-hydrogen atoms 1501

Macromolecules 1320

Ligands 72

Solvent 109

Protein residues 170

R.M.S. Deviations

Bonds (Å) 0.123

Angles (º) 1.77

Ramachandran map (%)

Favored 98.81

Allowed 1.19

Outliers 0.00

Rotamer outliers (%) 0.00

Clashscore 5.17

Average B-factor (Å^2^) 24.27

Macromolecules 23.98

Ligands 22.41

Solvent 29.05

**Table S1: Data collection and refinement statistics for the KRAS^G12C^ • divarasib complex.** Values in parentheses represent the highest resolution shell.

**Figure S1: Conformation of key switch-II pocket residues in complex with each KRAS^G12C^ drug.** Illustrations were generated in PyMOL**.** Distances are shown in Å. Reported crystal structures are less than 2.0 Å resolution **(A.)** Key residues that interact with sotorasib include His 95 and Tyr 96 that allow space for the isopropyl-methylpyridine. Taken from PDB: 6OIM. **(B.)** Key residues interacting with adagrasib include Glu 62 which reaches over the quinazoline core to form an interaction with the nitrogen of the methylpyrrolidine. Taken from PDB: 6UT0. **(C.)** Switch-II pocket residues adjacent to opnurasib. His 95 and Tyr 96 notably are further from the drug while the main chains of the switch-II loop such as Ser 65 form interactions. Taken from PDB: 7R0M. **(D.)** BI-0474 in the switch-II pocket. The buried benzothiophene group is 2.8 Å from Asp 69 forming a hydrogen bond. Three water molecules sit behind the drug to form a network with Gly 10, Lys 16, Thr 58, and Tyr 96. Taken from PDB: 8AFB.

**Figure S2: Electron density map for the switch-II region of the KRAS^G12C^ • divarasib complex.** Residues 58 – 72 are shown in two different views. The 2Fo-Fc map is contoured to 1σ.
